# QSPR/QSAR study of antiviral drugs modeled as multigraphs by using TI’s and MLR method to treat COVID-19 disease

**DOI:** 10.1038/s41598-024-63007-w

**Published:** 2024-06-07

**Authors:** Ugasini Preetha P, M. Suresh, Fikadu Tesgera Tolasa, Ebenezer Bonyah

**Affiliations:** 1 Department of Mathematics, College of Engineering and Technology, SRM Institute of Science and Technology, Kattankulathur, Tamil Nadu 603203 India; 2 Department of Mathematics, Dambi Dollo University, Oromia, Ethiopia; 3 Department of Mathematics Education, Akenten Appiah Menka University of Skills Training and Entrepreneurial Development, Kumasi, Ghana

**Keywords:** Antiviral drugs, M-polynomial, NM-polynomial, QSAR/QSPR, Molecular multigraphs, Multiple linear regression, Biotechnology, Cancer, Chemistry, Mathematics and computing, Nanoscience and technology

## Abstract

The ongoing COVID-19 pandemic continues to pose significant challenges worldwide, despite widespread vaccination. Researchers are actively exploring antiviral treatments to assess their efficacy against emerging virus variants. The aim of the study is to employ M-polynomial, neighborhood M-polynomial approach and QSPR/QSAR analysis to evaluate specific antiviral drugs including Lopinavir, Ritonavir, Arbidol, Thalidomide, Chloroquine, Hydroxychloroquine, Theaflavin and Remdesivir. Utilizing degree-based and neighborhood degree sum-based topological indices on molecular multigraphs reveals insights into the physicochemical properties of these drugs, such as polar surface area, polarizability, surface tension, boiling point, enthalpy of vaporization, flash point, molar refraction and molar volume are crucial in predicting their efficacy against viruses. These properties influence the solubility, permeability, and bio availability of the drugs, which in turn affect their ability to interact with viral targets and inhibit viral replication. In QSPR analysis, molecular multigraphs yield notable correlation coefficients exceeding those from simple graphs: molar refraction (MR) (0.9860), polarizability (P) (0.9861), surface tension (ST) (0.6086), molar volume (MV) (0.9353) using degree-based indices, and *flash point (FP)* (0.9781), surface tension (ST) (0.7841) using neighborhood degree sum-based indices. QSAR models, constructed through multiple linear regressions (MLR) with a backward elimination approach at a significance level of 0.05, exhibit promising predictive capabilities highlighting the significance of the biological activity $IC_{50}$ (Half maximal inhibitory concentration). Notably, the alignment of predicted and observed values for Remdesivir’s with obs ${pIC_{50} = 6.01}$,pred ${pIC_{50} = 6.01}$ ($pIC_{50}$ represents the negative logarithm of $IC_{50}$) underscores the accuracy of multigraph-based QSAR analysis. The primary objective is to showcase the valuable contribution of multigraphs to QSPR and QSAR analyses, offering crucial insights into molecular structures and antiviral properties. The integration of physicochemical applications enhances our understanding of factors influencing antiviral drug efficacy, essential for combating emerging viral strains effectively.

## Introduction

Graph theory has seen a surge in its application to pharmacology and medicine, with chemical graph theoreticians focusing on computing topological indices of drug structures to gain insights into molecular properties and aid in drug development. SARS-CoV-2, a single-stranded RNA virus, causes COVID-19, the first major pandemic of the twenty-first century. In 2003, SARS, caused by a new corona virus strain, led to 916 deaths globally. Similarly, COVID-19 emerged in December 2019, originating in Wuhan, China, and was declared a global public health emergency by the WHO in January 2020^1^. We are in the half past of 2023, but still, we are facing the corona virus pandemic situation. As of May 12, 2024, 10:39am CEST, the World Health Organization (WHO) has reported a global total of 775,379,864 confirmed COVID-19 cases, with 7 million recorded fatalities. For the latest statistics, refer to https://covid19.who.int/.

Our research, extending on prior studies highlighting double bonds, could improve correlation results in molecular modeling. Our study is inspired by previous research such as that by Kier et al.’s^2^ observation in “Medicinal Chemistry: A Series of Monographs” about double-edge counts providing a more accurate representation of double bonds. Recent work by Simon et al. also indicated improved correlations for molecules with weighted Wiener indices compared to traditional Wiener indices for simple graphs, while Zakharov et al. proposed a novel approach using multigraphs for enhanced statistical QSAR model building^3,4^. Using these insights, by these insights, we conducted a comparative analysis between simple and complex models to investigate the impact of double bonds on property estimation accuracy. Topological indices analyze the structure-property relationships in chemical compounds, providing numerical parameters for QSPR and QSAR studies. The research on TI’s has led to the development of over 3000 indices, reflecting the structural properties of the graphs used for their calculation. Most recently, Sakander Hayat et al. research explores the use of temperature-based topological indices, valency-based descriptors, distance-based graphical indices, and eigenvalues-based indices to predict physicochemical and thermodynamic properties of polycyclic aromatic hydrocarbons and benzenoid hydrocarbons^5–10^. Recently, QSPR/QSAR analysis on the antiviral drugs, corona drugs and anticancer drugs has been analyzed using degree/reverse degree/distance/neighborhood based topological descriptors^11–16^. Zaman et al.^17–26^ research delves into diverse applications of analytical and theoretical studies in chemistry and related fields, focusing on structural analysis, topological characterization, and mathematical modeling of various nanostructures, biochemical networks, and metal-organic models. The author’s work explores the relationships between molecular topology, irregular molecular descriptors, and novel topological indices, offering insights into the structural properties of complex materials and nanostructures.

This article represents chemical structures using hydrogen suppressed molecular multigraphs with the inclusion of double bonds. A multigraph is a graph containing multiple edges, where multiple edges indicate more than one connection between two vertices, and loops represent edges connecting the same vertex at both ends^27^. Marrero Ponce in^28^ discusses the application of QSPR/QSAR analysis for pseudo-graphs (graphs with loops and parallel edges), with considerations for hetero-atoms using the Valence delta concept^29^. This study compares multigraph and simple graph modeling approaches using topological structure descriptors to estimate physicochemical and biological activity through QSPR/QSAR analysis. Multiple linear regression techniques validate correlation values, aiding in understanding estimators and identifying potential drugs. Notably, no previous literature directly compares multigraph and simple graph efficacy in this context, making this study’s contribution novel and original.

In this study, multigraphs are employed to establish correlations between the physicochemical properties and biological activity of the antiviral drugs. Our QSAR model, utilizing multigraphs, demonstrates a stronger association between the studied biological activity $(pIC_{50})$ with the topological indices compared to the QSAR model proposed by Kirmani et al.^11^. Scientific literature has introduced several graph polynomials to aid in the calculation of various graph indices. Distance-based polynomials like the Hosoya polynomial, PI polynomial, Schultz polynomial, and modified Schultz polynomial have been suggested in previous studies see^30–32^. In addition, Deutsch and Klavzar (2015)^33^ developed the M-polynomial as a means to compute different degree-based TI’s.

The M-polynomial of graph $\mathscr {G}$ is defined in the following manner

1 $$\begin{aligned} M(\mathscr {G};x,y) = \sum _{j \le k}^{{\phantom{i}}} m_{jk}(\mathscr {G})x^jy^k \end{aligned}$$

In this context, $m_{jk}$ represents the count of edges uv $\in$ $E(\mathscr {G})$, where $d_u$ and $d_v$ are the degrees of vertices u and v, respectively, and (j, k) corresponds to their respective degrees. The NM-polynomial, akin to the M-polynomial, is a polynomial designed specifically for neighborhood degree sum-based indices^34^. It serves a similar purpose and its definition is as follows:

2 $$\begin{aligned} NM^{*}(\mathscr {G};x,y) = \sum _{j \le k}^{{\phantom{i}}} nm^{*}_{jk}(\mathscr {G})x^jy^k \end{aligned}$$

Here $nm^{*}_{jk}$ represents the count of edges uv $\in$ $E(\mathscr {G})$, where $nd^{*}_u$, $nd^{*}_v$ = (j,k) respectively. $nd^{*}_u$, $nd^{*}_v$ denotes the neighborhood degree of the vertices u and v in the graph respectively. The objective of this research is to create reliable QSPR/QSAR models that can effectively forecast the physical/chemical and biological properties of drugs targeting COVID-19. Throughout the article, the abbreviations ‘NBD’ (neighborhood degree sum-based indices) and ’D’ (Degree based indices) are used in specific sections for convenience.

## Material and method

In our study, we utilized algebraic polynomials to determine the topological indices of several antiviral drugs’ structures, our analysis yielded important findings in this regard. Table 1 presents the relationship between different TI’s derived from the M-polynomial and NM-polynomial and the range of integration defined in Table 1 as x = 1 and y = 1 is proved by Sandi Klavžar in^33^. Neighborhood degree sum-based topological indices, as discussed in references^35,36^ which demonstrates a remarkable capability to predict various physicochemical properties with high accuracy. Furthermore, a parallel effort has led to the construction of several other neighborhood degree sum-based topological indices, along with their corresponding classical degree-based topological indices, as detailed in references^37–39^. Mondal et al. conducted a study^28^ to assess the efficacy of four antiviral drugs in the treatment of COVID-19 patients. The study employed the M-polynomial and NM-polynomial methods for evaluation purposes. Additionally, Kirmani et al.^11^ recently developed QSPR/QSAR models utilizing linear and multiple linear regression to establish relationships between physicochemical/biological properties and potential antiviral drugs using TI’s in the context of COVID-19 treatment.

**Figure 1:**
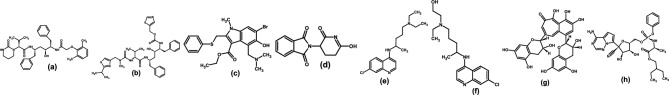
Chemical structures of (**a**) Lopinavir, (**b**) Ritonavir, (**c**) Arbidol, (**d**) Thalidomide, (**e**) Chloroquine, (**f**) Hydroxy-chloroquine, (**g**) Theaflavin, (**h**) Remdesivir.

To model the antiviral activity of drugs investigated for COVID-19 treatment, a combination of ten ’D’ and ten ’NBD’ based TI’s, alongside eight physicochemical properties, such as polar surface area, polarizability, surface tension, boiling point, enthalpy of vaporization, flash point, molar refraction and molar volume, were employed. The study focused on analyzing the drugs Hydroxychloroquine, Theaflavin, Lopinavir, Ritonavir, Arbidol, Chloroquine and Remdesivir. Thalidomide was excluded from the QSAR study due to insufficient available data on its antiviral activity. Fig. 1 displays the chemical structures of these drugs. We utilized *ChemSketch* to generate visual representations of the below chemical drug structures. Within this article, the QSAR model incorporates the biological activity $IC_{50}$ (Half maximal inhibitory concentration) to predict the antiviral activity of the mentioned drugs. Multiple linear regression (MLR) is employed as the statistical technique for this purpose. $IC_{50}$ is a widely used measure in drug development to assess the strength of potential drug candidates and compare their efficacy. It is also used in biochemical studies to understand the properties of proteins and enzymes. $pIC_{50}$ represents the negative logarithm of $IC_{50}$. The physicochemical properties and biological activity data of the antiviral drugs mentioned are presented in Table 2. These values were sourced from ChemSpider and the half-maximal inhibitory concentrations ($IC_{50}$) of antiviral activity for the compounds were collected from the scientific literature^11,40–43^. and converted to their negative logarithmic scale ($pIC_{50}$) to facilitate data analysis and interpretation.

**Table 1:** Description and derivation of various TI’s obtained from the M-polynomial and NM-polynomial where $D_x = x\bigg (\frac{\partial (z(x,y))}{\partial x}\bigg )$, $D_y = y\bigg (\frac{\partial (z(x,y))}{\partial y}\bigg )$, $S_x = \int _{0}^{x} \frac{z(t,y)}{t}dt$, $S_y = \int _{0}^{y} \frac{z(x,t)}{t}dt$, $J(z(x,y)) = z(x,x)$, $Q_{\alpha }(z(x,y)) = x^{\alpha }z(x,y)$ For D based TI’s: $\Delta (u)=d_u$, $\Delta (v) = d_v$, $z(x,y) = M(\mathscr {G};x,y)$ for NBD based TI’s: $\Delta (u)=nd^{*}_u$, $\Delta (v) = nd^{*}_v$, $z(x,y) = NM^{*}(\mathscr {G};x,y)$.

Topological index (TI)	Formula $g(\Delta (u), \Delta (v))$	Derivation from z(x,y) = $M(\mathscr {G};x,y)$ or $NM^{*}(\mathscr {G};x,y)$	References of TI’s first introduced:
$1^{st}$ Zagreb index: $M_1(\mathscr {G})$	$\sum _{uv \in E(\mathscr {G})}^{{\phantom{i}}} (\Delta (u) + \Delta (v))$	$(D_x + D_y)(z(x,y))_{x=y=1}$	^44^
$3^{rd}$ version Zagreb index: $NM_1^{*}(\mathscr {G})$			
$2^{nd}$ Zagreb index: $M_2(\mathscr {G})$	$\sum _{uv \in E(\mathscr {G})}^{{\phantom{i}}} (\Delta (u) \times \Delta (v))$	$(D_x \times D_y)(z(x,y))_{x=y=1}$	^44^
Nbd $2^{nd}$ zagreb index: $NM_2^{*}(\mathscr {G})$			
$2^{nd}$ modified Zagreb index: $mM_2(\mathscr {G})$	$\sum _{uv \in E(\mathscr {G})}^{{\phantom{i}}} \frac{1}{\Delta (u)\Delta (v)}$	$(S_x S_y)(z(x,y))_{x=y=1}$	^45^
Nbd $2^{nd}$ modified zagreb index: $NmM_2^{*}(\mathscr {G})$			
Redefined $3^{rd}$ Zagreb index: $ReZG_3(\mathscr {G})$	$\sum _{uv \in E(\mathscr {G})}^{{\phantom{i}}}[\Delta (u)\Delta (v) \times (\Delta (u)+\Delta (v))]$	$D_xD_y(D_x+D_y)(z(x,y))_{x=y=1}$	^46,47^
$3^{rd}$ Nde index: $ND_3^{*}(\mathscr {G})$			
Forgotten topological index: $F(\mathscr {G})$	$\sum _{uv \in E(\mathscr {G})}^{{\phantom{i}}} (\Delta ^{2}(u) + \Delta ^{2}(v))$	$(D_x^{2} + D_y^{2})(z(x,y))_{x=y=1}$	^48^
Nbd Forgotten topological index: $NF^{*}(\mathscr {G})$			
Randic index: $R_{\alpha }(\mathscr {G})$	$\sum _{uv \in E(\mathscr {G})}^{{\phantom{i}}} (\Delta (u) \Delta (v))^{\alpha }$	$(D_x^{\alpha } D_y^{\alpha })(z(x,y))_{x=y=1}$	^49^
Nbd Randic index: $NR_{\alpha }^{*}(\mathscr {G})$			
Inverse Randic index: $RR_{\alpha }(\mathscr {G})$	$\sum _{uv \in E(\mathscr {G})}^{{\phantom{i}}} \frac{1}{(\Delta (u)\Delta (v))^{\alpha }}$	$(S_x^{\alpha } S_y^{\alpha })(z(x,y))_{x=y=1}$	^50^
Nbd inverse Randic index: $NRR_{\alpha }^{*}(\mathscr {G})$			
Symmetric Division index: $SDD(\mathscr {G})$	$\sum _{uv \in E(\mathscr {G})}^{{\phantom{i}}} \frac{\Delta ^{2}(u) + \Delta ^{2}(v)}{\Delta (u)\Delta (v)}$	$(D_xS_y + S_x D_y)(z(x,y))_{x=y=1}$	^51^
Fifth NDe index: $ND_5^{*}(\mathscr {G})$			
Harmonic index: $H(\mathscr {G})$	$\sum _{uv \in E(\mathscr {G})}^{{\phantom{i}}} \frac{2}{\Delta (u)+\Delta (v)}$	$(2S_xJ)(z(x,y))_{x=1}$	^52^
Nbd Harmonic index: $NH^{*}(\mathscr {G})$			
Inverse sum indeg index: $I(\mathscr {G})$	$\sum _{uv \in E(\mathscr {G})}^{{\phantom{i}}} \frac{\Delta (u)\Delta (v)}{\Delta (u)+\Delta (v)}$	$(S_xJD_xD_y)(z(x,y))_{x=1}$	^51^
Nbd Inverse sum indeg index: $NI^{*}(\mathscr {G})$			
Augmented Zagreb index: $A(\mathscr {G})$	$\sum _{uv \in E(\mathscr {G})}^{{\phantom{i}}} \big \{\frac{\Delta (u)\Delta (v)}{\Delta (u)+\Delta (v)-2}\big \}^{3}$	$(S_x^{3}Q_{-2}JD_x^{3}D_y^{3})(z(x,y))_{x=1}$	^53,54^
Sanskurti index: $S^{*}(\mathscr {G})$			

**Table 2:** Physicochemical properties/biological activity of several antiviral drugs.

Drugs	$BP (^{\circ } \text {C})$	$E (\text {kJ/mol})$	$FP (^\circ \text {C})$	$MR (\text {cm}^3)$	*PSA* (Å$^{2}$)	$P (\text {cm}^3)$	$T (\text {dyne/cm})$	$MV (\text {cm}^3)$	$IC_{50} (\mu M)$	$pIC_{50} (M)$
Lopinavir	924.2	140.8	512.7	179.2	120	71	49.5	540.5	5.25	5.28
Ritonavir	947	144.4	526.6	198.9	202	78.9	53.7	581.7	8.63	5.06
Arbidol	591.8	91.5	311.7	121.9	80	48.3	45.3	347.3	3.54	5.45
Thalidomide	487.8	79.4	248.8	65.2	87	25.9	71.6	161	-	-
Chloroquine	460.6	72.1	232.3	97.4	28	38.6	44	287.9	1.38	5.86
Hydroxy-chloroquine	516.7	83	266.3	99	48	39.2	49.8	285.4	0.72	6.14
Theaflavin	1003.9	153.5	336.5	137.3	218	54.4	138.6	301	8.44	5.07
Remdesivir	-	-	-	149.5	213	59.3	62.3	409	0.99	6.01

## Results and discussions

### Computation of M-polynomial and NM-polynomial of Lopinavir

**Figure 2:**
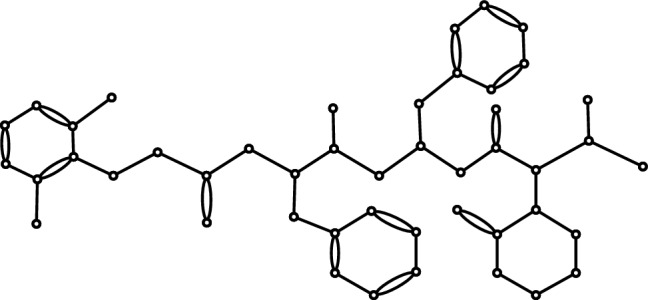
Molecular multigraph of Lopinavir.

**Figure 3:**
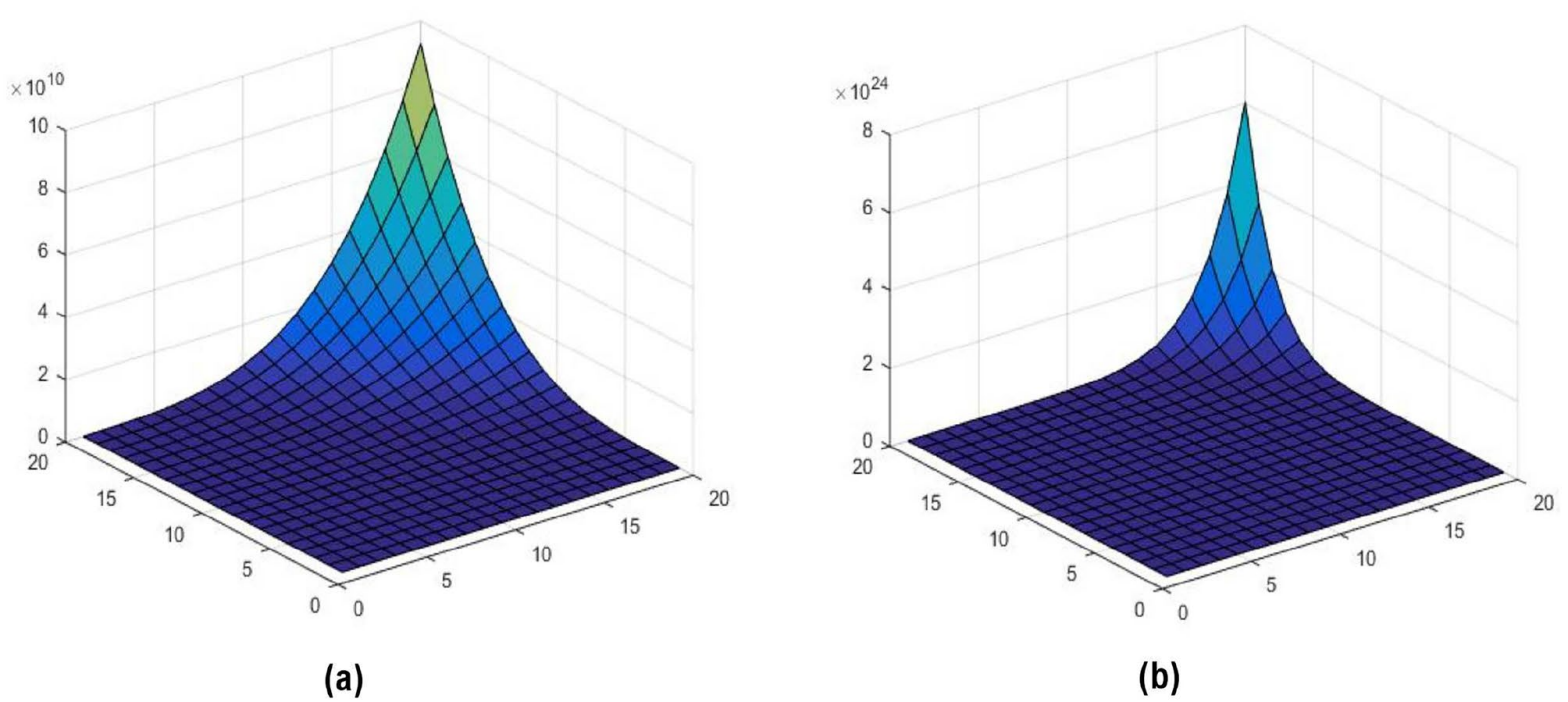
3D-plot generation of (**a**) M-polynomial and (**b**) NM-polynomial of Lopinavir.

In this section, we present the significant computational findings of our study. Our focus was on analyzing the molecular multigraph of lopinavir and deriving its M-polynomial and NM-polynomial, as described in the theorem below. Subsequently, we expanded our analysis to encompass seven additional molecular drug structures. We performed calculations to obtain the M-polynomial and NM-polynomial equations for each structure, and their corresponding values can be found in Table 3. Only lopinavir computation part is shown and Fig. 2 shows molecular multigraph of lopinavir. Figure 3 shows the 3D-Plot of M-polynomial and NM-polynomial of Lopinavir. From this observation the differences in the surface patterns imply that the degree-based and neighborhood degree-based topological indices derived from these polynomials will also differ in their numerical values and interpretations. To determine the superiority of one index over another, further analysis is required, such as comparing their performance in QSPR/QSAR models, evaluating their correlation coefficients with experimental data, and assessing their ability to discriminate between different molecular structures.

#### Theorem 1

*Let* $\mathscr {L}$ *be the molecular multigraph of Lopinavir. Then we have*,

$$\begin{aligned} M(\mathscr {L};x,y)= & {} 3xy^{3}+2xy^{4}+4x^{2}y^{2}+7x^{2}y^{3}+13x^{2}y^{4}+18x^{3}y^{3}+11x^{3}y^{4}+3x^{4}y^{4}\\ NM^{*}(\mathscr {L};x,y)= & {} 2x^{3}y^{5}+x^{3}y^{6}+x^{4}y^{4}+x^{4}y^{5}+3x^{4}y^{6}+4x^{4}y^{7}+2x^{4}y^{8}+x^{5}y^{9}+x^{5}y^{10}+10x^{6}y^{6}+14x^{6}y^{7}+x^{6}y^{10}+3x^{7}y^{7}+11x^{7}y^{8}+x^{7}y^{9}+x^{7}y^{10}+3x^{8}y^{10}+x^{9}y^{10} \end{aligned}$$

#### Proof

Consider $\mathscr {L}$ as the molecular multigraph representing Lopinavir (refer to Fig. 2). It comprises a total of 61 edges. Let $\Gamma _{(j,k)}$ represent the collection of edges where the endpoints have degrees i and j, respectively. (i.e.) $\Gamma _{(j,k)} = \{uv \in E(\mathscr {L}): \Delta (u) = j, \Delta (v) = k \}$. Let $m_{(i,j)}$ be the no.of edges in $\Gamma _{(j,k)}$. From 2 it is clear that $m_{(1,3)} = 3, m_{(1,4)} = 2, m_{(2,2)} = 4, m_{(2,3)} = 7, m_{(2,4)} = 13, m_{(3,3)} = 18, m_{(3,4)} = 11, m_{(4,4)} = 3$. To derive the M-polynomial of G, we use Eq. 1.

$$\begin{aligned} \begin{aligned} M(\mathscr {L};x,y)&= \sum _{j \le k}^{} m_{(j,k)}x^jy^k \\&= m_{(1,3)}x^{1}y^{3}+ m_{(1,4)}x^{1}y^{4}+ m_{(2,2)}x^{2}y^{2} + m_{(2,3)}x^{2}y^{3}+ m_{(2,4)}x^{2}y^{4} + m_{(3,3)}x^{3}y^{3} + m_{(3,4)}x^{3}y^{4}+ m_{(4,4)}x^{4}y^{4}. \end{aligned} \end{aligned}$$

By using the values of $m_{(j,k)}$, we get

$$\begin{aligned} M(\mathscr {L};x,y) = 3xy^{3}+2xy^{4}+4x^{2}y^{2}+7x^{2}y^{3}+13x^{2}y^{4}+18x^{3}y^{3}+11x^{3}y^{4}+3x^{4}y^{4}. \end{aligned}$$

Let $\Gamma ^{*}_{(j,k)}$ as the set of all edges in which the neighborhood degree sum of the endpoints corresponds to degrees i and j, respectively. (i.e.,) $\Gamma ^{*}_{(j,k)} = \{uv \in E(\mathscr {L}): \Delta (u) = j, \Delta (v) = k \}$. Let $nm^{*}_{(i,j)}$ be the no.of edges in $\Gamma ^{*}_{(j,k)}$. From 2 it is clear that $nm^{*}_{(3,5)} = 2, nm^{*}_{(3,6)} = 1, nm^{*}_{(4,4)} = 1, nm^{*}_{(4,5)} = 1, nm^{*}_{(4,6)} = 3, nm^{*}_{(4,7)} = 4, nm^{*}_{(4,8)} = 2, nm^{*}_{(5,9)} = 1, nm^{*}_{(5,10)} = 1, nm^{*}_{(6,6)} = 10, nm^{*}_{(6,7)} = 14, nm^{*}_{(6,10)} = 1, nm^{*}_{(7,7)} = 3, nm^{*}_{(7,8)} = 11, nm^{*}_{(7,9)} = 1, nm^{*}_{(7,10)} = 1, nm^{*}_{(8,10)} = 3, nm^{*}_{(9,10)} = 1$. To derive the NM-polynomial of G, we use Eq. (2).

$$\begin{aligned} NM^{*}(\mathscr {L};x,y)= & {} \sum _{j \le k}^{} nm^{*}_{(j,k)}x^jy^k \\= & {} nm^{*}_{(3,5)}x^{3}y^{5}+ nm^{*}_{(3,6)}x^{3}y^{6}+ nm^{*}_{(4,4)}x^{4}y^{4}+ nm^{*}_{(4,5)}x^{4}y^{5}+ nm^{*}_{(4,6)}x^{4}y^{6} + nm^{*}_{(4,7)}x^{4}y^{7} + nm^{*}_{(4,8)}x^{4}y^{8} \\{} & {} +nm^{*}_{(5,9)}x^{5}y^{9}+nm^{*}_{(5,10)}x^{5}y^{10}+nm^{*}_{(6,6)}x^{6}y^{6}+nm^{*}_{(6,7)}x^{6}y^{7}+nm^{*}_{(6,10)}x^{6}y^{10} +nm^{*}_{(7,7)}x^{7}y^{7} + nm^{*}_{(7,8)}x^{7}y^{8} \\{} & {} +nm^{*}_{(7,9)}x^{7}y^{9}+nm^{*}_{(7,10)}x^{7}y^{10}+nm^{*}_{(8,10)}x^{8}y^{10}+nm^{*}_{(9,10)}x^{9}y^{10}. \end{aligned}$$

The M-polynomial and NM-polynomial are computed to derive a range of ’D’ and ’NBD’ TI’s for the molecular multigraph representing Lopinavir. These findings are summarized in the following theorem. $\square$

#### Theorem 2

*Let* $\mathscr {L}$ *be the molecular multigraph of Lopinavir. Then, their respective values in Table* 3*holds*.

#### Proof

Initially, we determine the degree-based indices by referring to Table 1. Let $M(\mathscr {L};x,y) = t(x,y) = 3xy^{3}+2xy^{4}+4x^{2}y^{2}+7x^{2}y^{3}+13x^{2}y^{4}+18x^{3}y^{3}+11x^{3}y^{4}+3x^{4}y^{4}$. Then we have,

1. $$M_1(\mathscr {L}) = (D_x+D_y)t(x,y)|_{x=y=1} =12xy^{3}+10xy^{4}+16x^{2}y^{2}+35x^{2}y^{3}+78x^{2}y^{4}+108x^{3}y^{3}+77x^{3}y^{4} +24x^{4}y^{4} = 360.$$
2. $$M_2(\mathscr {L}) = (D_xD_y)t(x,y)|_{x=y=1} = 9xy^{3}+8xy^{4}+16x^{2}y^{2}+42x^{2}y^{3}+104x^{2}y^{4}+162x^{3}y^{3}+132x^{3}y^{4}+48x^{4}y^{4}$$
3. $$mM_2(\mathscr {L}) = S_xS_yt(x,y)|_{x=y=1} = xy^{3}+\frac{2}{4}xy^{4}+x^{2}y^{2}+\frac{7}{6}x^{2}y^{3}+\frac{13}{8}x^{2}y^{4}+\frac{18}{9}x^{3}y^{3}+\frac{11}{12}x^{3}y^{4}+\frac{3}{16}x^{4}y^{4} = 8.3958$$
4. $$ReZG_3(\mathscr {L}) = D_xD_y(D_x+D_y)t(x,y)|_{x=y=1} = 36xy^{3}+40xy^{4}+64x^{2}y^{2}+210x^{2}y^{3}+624x^{2}y^{4}+972x^{3}y^{3}+924x^{3}y^{4}+384x^{4}y^{4} = 3254$$
5. $$F(\mathscr {L}) = (D_x^{2}+D_y^{2})t(x,y)|_{x=y=1} = 30xy^{3}+34xy^{4}+32x^{2}y^{2}+91x^{2}y^{3}+260x^{2}y^{4}+324x^{3}y^{3}+275x^{3}y^{4}+96x^{4}y^{4} = 1142$$
6. $$SDD(\mathscr {L}) = (S_xD_y+S_yD_x)t(x,y)|_{x=y=1} = \frac{30}{3}xy^{3}+\frac{34}{4}xy^{4}+\frac{32}{4}x^{2}y^{2}+\frac{91}{6}x^{2}y^{3}+\frac{260}{8}x^{2}y^{4}+\frac{324}{9}x^{3}y^{3} +\frac{275}{12}x^{3}y^{4}+ \frac{96}{16} = 139.0833$$
7. $$H(\mathscr {L}) = 2S_xJt(x,y)|_{x=1} = \frac{7}{4}x^{4}+\frac{9}{5}x^{5}+\frac{31}{6}x^{6}+\frac{11}{7}x^{7}+\frac{3}{8}x^{8} = 21.3262$$
8. $$I(\mathscr {L}) = S_xJD_xD_yt(x,y)|_{x=1} = \frac{25}{4}x^{4}+\frac{50}{5}x^{5}+\frac{266}{6}x^{6}+\frac{132}{7}x^{7}+\frac{48}{8}x^{8} = 85.4405$$
9. $$A(\mathscr {L}) = S_x^{3}Q_{-2}JD_x^{3}D_y^{3}t(x,y)|_{x=1} = 42.125x^{2}+60.7407x^{3}+309.0313x^{4}+152.064x^{4}+56.8889x^{6} = 620.8499$$
10. $$R_{\alpha }(\mathscr {L}) = D_x^{\alpha }D_y^{\alpha }t(x,y)|_{x=1} 3(3)^{\alpha }+2(4)^{\alpha }+4(4)^{\alpha }+7(6)^{\alpha }+13(8)^{\alpha }+18(9)^{\alpha }+11(12)^{\alpha }+3(16)^{\alpha } = 22.1114$$

Next, we compute the neighborhood degree sum-based indices by taking into account $NM^{*}(\mathscr {L}) = t(x,y) = 2x^{3}y^{5}+x^{3}y^{6}+x^{4}y^{4}+x^{4}y^{5}+3x^{4}y^{6}+4x^{4}y^{7}+2x^{4}y^{8}+x^{5}y^{9}+x^{5}y^{10}+10x^{6}y^{6}+14x^{6}y^{7}+x^{6}y^{10}+3x^{7}y^{7}+11x^{7}y^{8}+x^{7}y^{9}+x^{7}y^{10}+3x^{8}y^{10}+x^{9}y^{10}$. By utilizing the edge partition of $\Gamma ^{*}_{(j,k)}$ in combination with Table 1, the NM-polynomial can be derived, thus concluding the proof. The obtained values of the ’D’ & ’NBD’ indices, calculated using the M-polynomial and NM-polynomial, are displayed in Tables 3 and 4, respectively. $\square$

**Table 3:** Selected antiviral drugs with degree based TI’s.

Drugs	$M_1$	$M_2$	$mM_2$	$ReZG_3$	*F*	*SDD*	*H*	*I*	*A*	$R_{-1/2}$
Lopinavir	360	521	8.3958	3254	1142	139.0833	21.3262	85.4405	620.8499	22.1114
Ritonavir	388	555	9.1388	3418	1224	151	23.0714	91.7619	651.0746	23.9889
Arbidol	246	391	5.375	2708	846	90.8333	13.1524	58.4309	463.4311	13.7781
Thalidomide	178	276	3.125	1830	602	62.0833	8.9952	42.438	317.3156	9.2407
Chloroquine	164	245	4.4444	1586	524	62.75	10.2571	39.3595	307.4737	10.6064
Hydroxy-chloroquine	168	249	4.6944	1602	532	64.75	10.7571	40.3595	315.4737	11.1064
Theaflavin	376	610	6.6736	4280	1332	135.6666	17.869	88.8142	697.1812	18.8432
Remdesivir	340	508	7.8402	3300	1126	128.5833	19.3833	80.3214	598.6463	20.1467

**Table 4:** Selected antiviral drugs with neighborhood degree sum based TI’s.

Drugs	$NM_1$	$NM_2$	$NmM_2$	$NDe_3$	*NF*	$NDe_5$	*NH*	*NI*	*S*	$NR_{-1/2}$
Lopinavir	800	2661	1.6381	37260	5528	127.8742	9.6532	195.9077	3591.3877	9.7787
Ritonavir	844	2732	1.8493	36800	5628	137.9468	10.6676	207.326	3646.3337	10.8076
Arbidol	586	2340	1.0579	40974	4896	83.8	5.813	142.4999	3579.5211	5.9331
Thalidomide	420	1621	0.6197	27492	3447	217.3108	3.9576	101.2374	2392.9166	4.0512
Chloroquine	376	1348	0.9201	21056	2802	59.1424	4.6471	91.9638	1932.3384	4.7313
Hydroxy-chloroquine	382	1353	1.0276	20960	2812	60.9646	4.9691	93.5058	1932.7098	5.0463
Theaflavin	914	3721	1.0495	63870	7726	121.7193	7.3926	223.3278	5627.8009	7.5288
Remdesivir	782	2816	1.5219	41542	5982	202.422	8.9074	179.9093	3770.4423	8.8021

### QSPR analysis of selected antiviral drugs with its target properties

#### Regression analyses

**Table 5:** Correlation coefficients (r) of degree based indices and the physicochemical properties of antiviral drugs modeled as molecular multigraphs using linear regression model.

Index	*BP*	*E*	*FP*	*MR*	*PSA*	*P*	*ST*	*MV*
$M_1$	0.9863	0.9826	0.8587	0.8895	0.8934	0.8897	0.3945	0.7534
$M_2$	0.9807	0.9778	0.7703	0.8216	0.908	0.8216	0.5083	0.6969
$mM_2$	0.8824	0.8725	0.9553	${\textbf {0.9860}}^{{\textbf {*}}}$	0.737	${\textbf {0.9861}}^{{\textbf {*}}}$	0.0404	${\textbf {0.9353}}^{{\textbf {*}}}$
$ReZG_3$	0.9483	0.9462	0.6592	0.7328	0.8988	0.7328	${\textbf {0.6086}}^{{\textbf {*}}}$	0.5435
*F*	0.9814	0.9788	0.7769	0.824	**0.9138**	0.8242	0.5009	0.6608
*SDD*	0.9773	0.9722	0.8994	0.9283	0.8661	0.9285	0.3141	0.8118
*H*	0.9307	0.9238	**0.9589**	0.9723	0.7975	0.9725	0.1447	0.8985
*I*	**0.9864**	**0.9827**	0.8608	0.8915	0.8909	0.8917	0.3916	0.7564
*A*	0.9851	0.9811	0.7953	0.8504	0.8949	0.8504	0.4678	0.6952
$R_{-1/2}$	0.9363	0.9293	0.9535	0.9712	0.8039	0.9714	0.1597	0.8932

Bolded values represent the highly correlated values for multigraph and * represent highly correlated values for multigraph compared to simple graph.

**Table 6:** Correlation coefficients (r) between ‘NBD’ and the physicochemical properties of antiviral drugs, modeled as molecular multigraphs using a linear regression model.

Index	*BP*	*E*	*FP*	*MR*	*PSA*	*P*	*ST*	*MV*
$NM_1$	0.9845	0.9816	0.7862	0.8322	**0.9094**	0.8323	0.4899	0.6721
$NM_2$	0.9157	0.9146	0.5803	0.6613	0.8904	0.6612	0.6661	0.4583
$NmM_2$	0.728	0.7168	0.9513	0.9638	0.5887	0.964	0.2036	**0.9755**
$NDe_3$	0.7684	0.7688	0.3337	0.4358	0.7822	0.4356	${\textbf {0.7841}}^{{\textbf {*}}}$	0.2085
*NF*	0.91	0.909	0.5715	0.6501	0.8946	0.6501	0.6664	0.4464
$NDe_5$	0.194	0.2146	0.1972	0.0534	0.5369	0.0551	0.2403	0.0169
*NH*	0.8857	0.8782	${\textbf {0.9781}}^{{\textbf {*}}}$	0.9781	0.758	0.9784	0.0446	0.9285
*NI*	**0.9858**	**0.9828**	0.7902	0.839	0.889	0.839	0.4949	0.6802
*S*	0.8439	0.8433	0.4498	0.5446	0.8265	0.5444	0.7427	0.3261
$NR_{-1/2}$	0.8873	0.8798	0.9777	**0.9818**	0.7523	**0.9821**	0.0492	0.9321

Bolded values represent the highly correlated values for multigraph and * represent highly correlated values for multigraph compared to simple graph.

**Figure 4:**
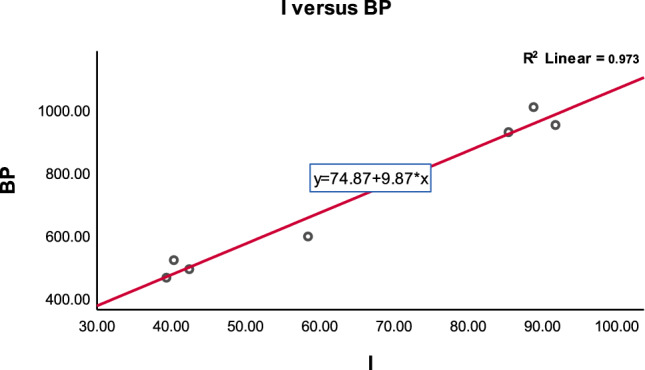
Inverse sum indeg index versus predicted boiling point.

To clarify the physical significance of our results, we have included concise discussions on the effectiveness of the computed topological indices. These quantitative measures reveal key structural attributes, with higher values indicating enhanced stability and lower reactivity, and lower values suggesting potential reactivity sites. Our study validates the predictive power of these indices by demonstrating strong correlations with experimental properties, supporting their use in understanding structure-property relationships and guiding drug design and development. We highlight the practical applications in drug delivery and material design while acknowledging the need to consider molecular context and explore advanced methods for improved accuracy.The correlated values between ‘D’ and ‘NBD’ based TI’s and the physicochemical properties of antiviral drugs (COVID-19 drugs) can be observed in Tables 5 and 6. From Table 5 we observe that inverse sum indeg index (estimator) reflects a strong positive relationship with boiling point(outcome variable) which is depicted in Fig. 4.

**Figure 5:**
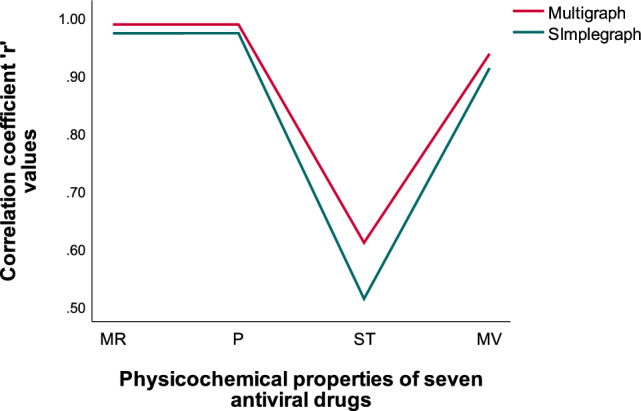
Comparison chart of ‘r’ values for multigraph versus simple graph: ‘D’.

**Figure 6:**
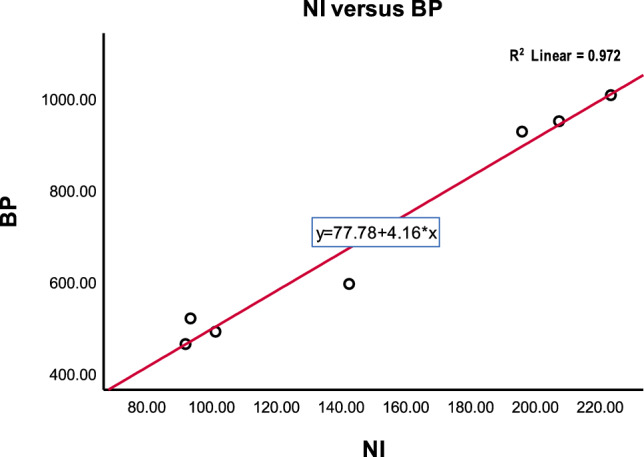
Neighborhood inverse sum indeg index versus predicted boiling point.

From Fig. 5 we observe that the high correlation coefficients ‘r’ values for the physicochemical properties of Surface tension(ST), Molar refractivity(MR), Molar volume(MV) and Polarizability(P) are higher than the simple graph’s representation of selected antiviral drugs. The existence of a double bond in a molecule can greatly impact its properties, including polarity, conjugation, and reactivity. These changes, in turn, can impact the molecule’s solubility, stability, and biological activity. For example when a molecule contains a double bond, it introduces regions of different electron density, resulting in a shift in polarity. The presence of the double bond can make the molecule more polar or less polar depending on the surrounding atoms and functional groups. We observe that molecular multigraphs can provide a more detailed and nuanced representation of the chemical structure and the high correlation coefficients ’r’ of simple graph representing seven drugs for the physicochemical properties of *MR with r = 0.9709, P = 0.9710, ST = 0.5115 and MV = 0.9108* using degree based indices from^11^. One can see the high correlation ‘r’ values of molecular multigraph in Table 5, bold values with an asterisk*. In similar fashion, From Table 6 we observe that Neighborhood Inverse sum indeg index(NI) (predictor variable) reflects a strong positive relationship with Boiling point(outcome variable) which is depicted in Fig. 6.

**Figure 7:**
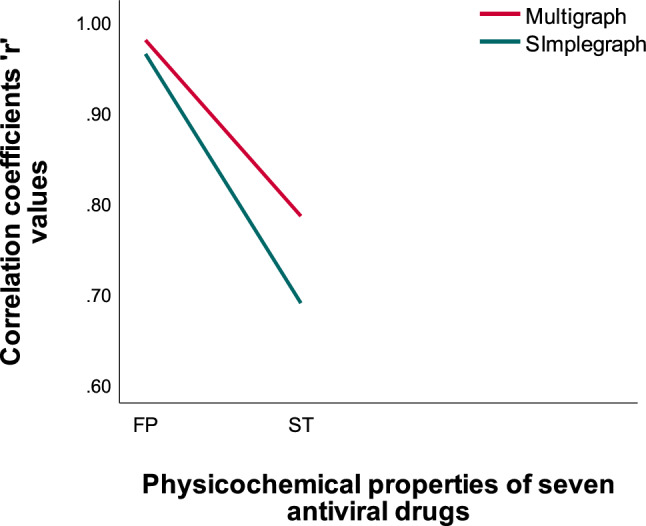
Comparison chart of ‘r’ values for multigraph versus simple graph: ‘NBD’.

From Fig. 7 we observe that the high correlation coefficients ’r’ values for the physicochemical properties of Flash point(FP) and Surface tension(ST) are higher than the simple graph’s representation of selected antiviral drugs. The high correlation coefficients ’r’ of simple graph representing seven drugs for the physicochemical properties of *FP with r = 0.9629 and ST with r = 0.6682* using Neighborhood degree sum based indices from^11^. One can see the high correlation ’r’ values of molecular multigraph in Table 6, bold values with an asterisk *.

Note: We also have observed that the highly correlated values in the multigraph are nearly identical to the values found in the simple graph for both ’D’ and ’NBD’ based correlation values for example, BP with 0.9920, E with 0.9887 from^11^ representing as simple graphs whereas for multigraphs BP with 0.9864 and E with 0.9827, we get a small variance with the correlation values and some are higher than the simple graph. However, when there is a low correlation between chemical structure descriptors and a target property, it suggests that additional factors may play a more significant role in determining the target property. Further analysis or experimentation might be necessary to identify and understand those factors.

### QSAR analyses of biological activity $pIC_{50}$ versus degree based & nbd degree sum-based indices as predictors

Within this section, we employed IBM SPSS Statistics Version 27.0.1.0 software. To view url link of this version, visit https://www.ibm.com/support/pages/downloading-ibm-spss-statistics-27010 to carry out multiple linear regression analyses. $IC_{50}$ were used as dependent variable and several ’D’ and ’NBD’ based indices, (one can refer Table 1) were used as independent variables. $IC_{50}$, also known as half maximal inhibitory concentration, is a parameter that measures the effectiveness of a drug or compound in inhibiting a specific biological or biochemical process. It represents the concentration at which the drug can block the target protein’s function by 50 %. $pIC_{50}$ is a transformed version of $IC_{50}$, where the “p” stands for the negative logarithm (base 10) of the $IC_{50}$ value. $pIC_{50}$ are used in regression analyses over $IC_{50}$ since it is linearly related to the drug potency than $IC_{50}$. The selection of the optimal multiple linear regression model was based on these statistical criteria: Fisher ratio (F), squared multiple correlation coefficient $(R^2)$, adjusted correlation coefficient $(R^{2}_{adj})$, Durbin–Watson value (DW), variance inflation factor (VIF), tolerance value and significance (Sig). The main difference between QSPR and QSAR is the type of property that is being predicted. QSPR models utilize statistical and mathematical methods to establish a link between the molecular structure of compounds and their physicochemical properties. On the other hand, QSAR models employ statistical and machine learning techniques to establish a correlation between the molecular structure of compounds and their biological activities.

#### MLR model and MLR analyses

Multiple linear regression (MLR)^55^ is a statistical technique that explores the relationship between a dependent variable and multiple independent variables. Its purpose is to find the best-fitting regression line that minimizes the differences between the predicted and actual values of the dependent variable. MLR is a statistical method that explores the linear relationship between target variable Y $(pIC_{50})$ and predictor variables X (2D descriptors). Through the least squares curve fitting technique, MLR calculates regression coefficients $(r^2)$ to estimate the model. This approach establishes a straight line equation that accurately represents the overall data points. The regression equation is formulated as follows:

3 $$\begin{aligned} Y = b_1 *I_1 + b_2 *I_2 + B_3 *I_3 + c \end{aligned}$$

In the regression equation, the dependent variable is represented as Y, and the regression coefficients ’b’ correspond to the independent variables ‘I’. The intercept or regression constant is denoted as ‘c’^56^. Kirmani et al.^11^ conducted a QSAR analysis on antiviral drugs represented as simple graphs, suggesting a weak association between biological activity $(pIC_{50})$ and TI’s. Inspired by their approach, we applied a similar analysis using molecular multigraphs for our selected drugs and achieved a well-fitting QSAR model by backward elimination method which will be elaborated in the upcoming section.

### Multicollinearity and VIF^57^

Multicollinearity refers to high correlation among independent variables, which can result in unstable and unreliable regression coefficient estimates. Variance inflation factor (VIF) is a measure used to evaluate the presence of multicollinearity in regression analysis, commonly utilized in tools such as SPSS and it is defined as $VIF = \frac{1}{1-R^2}$. VIF values ranging from 1 to 10 indicate no multicollinearity, while values below 1 or above 10 suggest the presence of multicollinearity. Our regression models showed signs of multicollinearity, as some independent variables had correlation coefficients near 1 and corresponding VIF values outside the ideal range of 1 to 10. This implies that the model may struggle to accurately estimate the individual effects of these correlated variables. Hence, it is crucial to address this issue to ensure the reliability and accuracy of our regression results.

### QSAR model for $pIC_{50}$

The correlation matrix is a helpful tool for detecting multicollinearity in regression models. It displays the pairwise correlations between multiple variables, indicating the strength and direction of their relationship. By examining the matrix for high correlations between independent variables, we can identify multicollinearity and take appropriate measures to address it. In the Supplementary Table S1, we present the correlation matrix between various ’D’ and ’NBD’ based indices. In QSAR analysis, one of the primary goals is to identify the most important molecular descriptors or features that are correlated with the target property. When dealing with numerous molecular descriptors in QSAR analysis, including all of them in the model may not be practical. To tackle this issue, variable selection techniques are utilized to identify the most significant descriptors that exhibit strong correlations with the target property. This process helps improve the predictive performance of the model. *Stepwise regression* is one such variable selection method that is commonly used in QSAR analysis. It involves iteratively adding or removing descriptors based on their statistical significance in predicting the target property. The process continues until no more significant descriptors remain, resulting in a effective model.

We began constructing simple linear regression models using topological indices that had the lowest correlation (specifically, 0.1170 between $NDe_3$ and $NmM_2$). This led to the development of two mono-parameter models. However, both models demonstrated a weak correlation with $pIC_{50}$.

Model 1 $$\begin{aligned} pIC_{50} = 6.183921-0.48734(\pm 0.502904)NmM_2 \end{aligned}$$

$$n=7, r=0.3976, R^2=0.1581, R_A^{2} = -0.01026, SE=0.4512, F=0.9390, PE=0.2121$$

Here n : Number of drugs used, r(R):simple(multiple) correlation coefficient, $R_A^{2}$: adjustable $R^{2}$, F: Fisher’s statistics, PE: Probability error.

By employing Stepwise regression analysis, various combinations of two topological indices have been examined. The following bi-parametric model demonstrates significantly improved statistical measures in comparison to its mono-parametric (Model 1).

Model 2 $$\begin{aligned} pIC_{50} = 6.782221-1.9E-05(\pm 1.06E-05)NDe_3-0.39912(\pm 0.422226)NmM_2 \end{aligned}$$

$$n=7, r=0.7292, R^2=0.5317, R_A^{2}=0.2976, SE=0.3762, F=2.2711, PE= 0.1179$$.

To improve the statistical parameters of the models, trials were conducted to determine the correlation between three combined TI’s and the biological activity$pIC_{50}$. However, the resulting model exhibited only marginal improvements in its statistical measures.

Model 3 $$\begin{aligned} pIC_{50} = 5.76991-0.00392(\pm 0.001944)S+0.000313(\pm 0.000165)NDe_3+1.170587(\pm 0.84103)NmM_2 \end{aligned}$$

$$n=7, r=0.8950, R^2=0.8011, R_A^{2}=0.6022, SE=0.2831, F=4.0282, PE= 0.0501$$.

By applying successive Stepwise regression, a tetra-parametric model was derived, showcasing notable enhancements in the statistical parameters.

Model 4 $$\begin{aligned} pIC_{50}&= 6.945062 + 0.001272(\pm 0.000599)NF - 0.00388(\pm 0.00132)S \\&\quad + 0.000167(\pm 0.00131)NDe_3 - 0.58105(\pm 1.003055)NmM_2 \end{aligned}$$

$$n=7, r=0.9689, R^2=0.9389, R_A^{2}=0.8167, SE=0.1921, F=7.6844, PE= 0.0154$$.

After employing successive Stepwise regression, a penta-parametric model was obtained, demonstrating enhanced statistical parameters.

Model 5 $$\begin{aligned} pIC_{50}&= 6.274774 + 0.030819(\pm 0.036622)NM_2 - 0.01093(\pm 0.014519)NF \\&\quad - 0.01637(\pm 0.014921)S + 0.000939(\pm 0.000928)NDe_3 + 0.726002(\pm 1.8948)NmM_2 \end{aligned}$$

$$n=7, r=0.9819, R^2=0.9642, R_A^{2}=0.7854, SE=0.2079, F=5.3922, PE= 0.0090$$.

In the aforementioned QSAR models, the F-value signifies the ratio between the variability accounted for by the model and the remaining variability ascribed to error. This value is used as an indicator of the model’s statistical significance, with a higher F-value suggesting a greater probability of statistical significance. Probability error, also known as a type I error or alpha error, refers to a statistical concept in hypothesis testing, $PE = \frac{2(1-r^2)}{3\sqrt{n}}$^56^. The p-value is a statistical measure that evaluates the likelihood of observing the given outcomes if the null hypothesis is true. It quantifies the level of evidence against the null hypothesis, indicating the strength of the observed results. A predetermined significance level, commonly set at 0.05, is used as a threshold to determine the statistical significance of the study findings and decide whether to reject the null hypothesis. In our QSAR models, we encountered insignificant results as our p (alpha) value was greater than 0.05. By selecting the least correlated variable can reduce the problem of pairwise correlations between the variables, it does not account for the possibility of higher-order correlations among the variables (multicollinearity). Pairwise correlation refers to the correlation between two variables. So we remove all the predictor variables included in the model since all our p values are greater than 0.05. To mitigate this problem, we used the backward elimination method. The objective was to identify a subset of predictor variables that exhibited the most robust association with the response variable $(pIC_{50})$ while avoiding the issue of over-fitting the model due to an excessive number of predictors.

#### Backward elimination method and validation

Backward elimination is a feature selection method used in statistical modeling and machine learning. It aims to identify the most relevant subset of features (independent variables) for a given predictive model. The method starts with a full model that includes all available features and iteratively eliminates features that are found to be non-significant. One can refer the article^58^ for QSAR study utilizing TI’s with backward elimination method. By conducting a 2D-QSAR analysis on the biological activity $pIC_{50}$ of antiviral drugs, we generated multiple QSAR models. During the stepwise regression process, we successfully identified and eliminated five independent variables that exhibited insignificant associations with the $pIC_{50}$ (biological activity) outcome. Initially, our study encompassed a total of 18 independent(predictor) variables, but after removing the insignificant features, we were left with 13 remaining predictors. The best linear model for $pIC_{50}$ contains three topological indices $ReZG_3, NDe_5$ and *NH*. Through the process of backward elimination, we initially considered all 13 predictors $M_1$, *F*, $M_2$, *H*, *SDD*, $mM_2$, *A*, *NH*, *I*, $NM_1$, $ReZG_3$, $NDe_5$ and *NI*. The aim was to identify the best subset of predictors(independent variables) that displayed a strong association with $pIC_{50}$. The selected model, **model 3** from **Table** 7, demonstrated the best combination of predictors based on various statistical parameters.

**Table 7:** Backward elimination: QSAR models.

Model		Unstandardized coefficients		Sig.	Collinearity statistics	
1		B	Std. error		Tolerance	VIF
	(Constant)	6.225	1.705	0.170		
	ReZG3	$-0.002$	0.003	0.602	0.001	981.421
	A	0.022	0.050	0.735	0.000	7253.512
	fifthNDe	0.008	0.014	0.668	0.019	53.041
	NH	$-0.411$	0.509	0.568	0.006	163.922
	NI	$-0.024$	0.118	0.873	0.000	4482.640
2	(Constant)	6.556	0.341	0.003		
	ReZG3	$-0.002$	0.002	0.431	0.001	970.311
	A	0.013	0.017	0.520	0.001	1632.970
	fifthNDe	0.010	0.003	0.059	0.255	3.921
	NH	$-0.415$	0.367	0.376	0.006	163.680
**3**	**(Constant)**	**6.746**	**0.220**	**0.000**		
	**ReZG3**	**0.000**	**0.000**	**0.020**	**0.440**	**2.273**
	**fifthNDe**	**0.009**	**0.002**	**0.022**	**0.342**	**2.924**
	**NH**	−**0.133**	**0.047**	**0.046**	**0.323**	**3.098**

The bolded values represent the significant model based on collinearity statistics.

### Validation: Durbin–Watson statistics and tolerance^59^

The Durbin–Watson statistic is used to measure autocorrelation in regression residuals. It ranges from 0 to 4, with 2 indicating no autocorrelation. Autocorrelation occurs when residuals are correlated over time, violating the assumption of independence. The DW statistic helps assess the level of correlation among residuals. A DW value below 2 indicates the presence of positive autocorrelation, while a value above 2 suggests negative autocorrelation. A DW value of 2 indicates the absence of autocorrelation. To evaluate the model’s goodness of fit using the Durbin-Watson (DW) statistic, a value close to 2 indicates no significant autocorrelation in the residuals. This suggests that the model effectively represents the relationship between the variables. In our final QSAR model 3, the DW value is around 2, indicating that the errors are uncorrelated. The concept of tolerance is employed as an indicator of multicollinearity, measuring the correlation among independent variables in a model. It is represented on a scale from 0 to 1, with a higher tolerance value nearing 1 indicating a lower degree of correlation among predictor variables, thus suggesting reduced multicollinearity. Conversely, a low tolerance value close to 0 indicates high correlation among predictors, suggesting a potential issue of multicollinearity.

## Discussion

Backward elimination typically uses a significance threshold (p-value) to determine whether a predictor should be removed from the model. If a predictor already exceeds the significance threshold at the beginning, it is considered non-significant and excluded directly without further evaluation. In our analysis, we found that 8 out of the 13 predictors did not meet the required statistical criteria, such as p-values, VIF, and tolerance values. As a result, these predictors were excluded from further analysis. The statistical parameters indicated that these predictors did not significantly contribute to the model and may have exhibited multicollinearity issues. So 5 independent predictors were carried out for backward elimination which is presented in Table 7, among which model 3 is the best to predict the biological activity $pIC_{50}$ based on these statistical criteria $VIF < 5$, Tolerance values are not close to zero, DW = 1.850 and all p-values are less than 0.05.

Model 3 $$\begin{aligned} pIC_{50} = 6.745589-0.133408(NH)+0.009435(NDe_5)-0.000442(ReZG_3) \end{aligned}$$

### Ordinary residuals or regular residuals^59^

**Figure 8:**
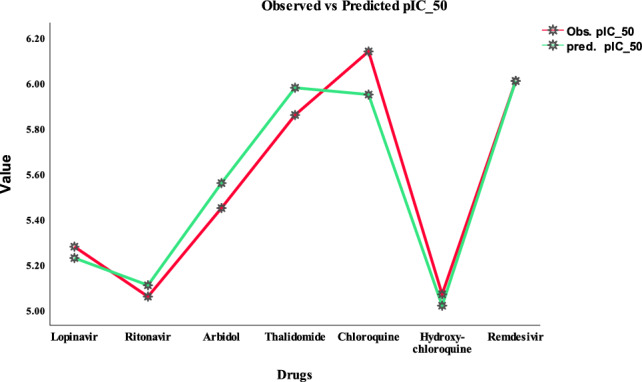
Comparison between observed and predicted values of $pIC_{50}$.

Regular Residual $=$ Observed Value − Predicted Value. In simpler words, a residual signifies the difference between the observed value of the dependent variable and the value estimated by a regression model. It represents the residual error or the remaining variability that the model was unable to explain. They measure the vertical difference between the observed data points and the regression line or curve. The comparison between the actual and independent (predicted) values of the biological activity $pIC_{50}$ for seven antiviral drugs is presented in Table 8. Figure 8 illustrates the linear relationship between the actual $pIC_{50}$ values and the predicted $pIC_{50}$ values obtained from model 3 for the aforementioned drugs.

**Table 8:** Comparison between predicted and observed values of model 3 for the validation of $pIC_{50}$ of the respective drugs.

Drugs	Obs. $pIC_{50}$	Pred. $pIC_{50}$	Res.
Lopinavir	5.28	5.23	0.05
Ritonavir	5.06	5.11	$-0.05$
Arbidol	5.45	5.56	$-0.11$
Chloroquine	5.86	5.98	$-0.12$
Hydroxy-chloroquine	6.14	5.95	0.19
Theaflavin	5.07	5.02	0.05
Remdesivir	6.01	6.01	0

## Conclusion

This study delves into the evaluation of various antiviral drugs for treating COVID-19, utilizing molecular multigraphs to analyze their chemical structures. Through edge partition techniques, M-polynomial and NM-polynomial expressions were derived, leading to the computation of ’D’ and ’NBD’ based indices. The research also involved a thorough QSPR investigation focusing on antiviral drugs as multigraphs, showcasing the predictive power of computed topological indices (TI’s) in determining physicochemical properties. Notably, the inverse sum indeg and neighborhood inverse sum indeg indices exhibited a strong positive correlation with boiling point (BP), surpassing other indices.

Further, QSAR analysis of the biological activity $pIC_{50}$ of these antiviral drugs were estimated using multiple linear regression in conjunction with backward elimination approach. The results demonstrated that the MLR model was an effective tool for estimating biological activity $pIC_{50}.$ The validation criteria used were designed to assess the accuracy and predictive capability of the MLR model. The results highlight the effectiveness of the MLR model in estimating $pIC_{50}$, with specific TI’s like *NH*, $NDe_5$, and $ReZG_3$ showing significant predictive potential. Also the observed and predicted $pIC_{50}$ of the drugs for the best model evaluated using cross validation techniques shows minor variation, resulting in low residuals.

The study highlights the importance of considering multigraphs as graph models, offering a novel perspective on drug connectivity analysis. By diverging from conventional approaches focused on simple graphs, the research has provided insights into optimizing the drug selection process. In conclusion, there remains an open challenge in incorporating chemometric methods statistical and mathematical techniques for analyzing chemical data to further refine these models. Using these techniques, researchers can advance our understanding of drug behavior and improve strategies for enhancing drug effectiveness.

## Supplementary Information

Supplementary Table S1.

## Data Availability

The paper includes the information used to verify the study’s findings.
